# Nano-Based Drug Delivery Systems: Potential Developments in the Therapy of Metastatic Osteosarcoma—A Narrative Review

**DOI:** 10.3390/pharmaceutics15122717

**Published:** 2023-12-01

**Authors:** Yuanrui Luo, Minghao Sun, Linyun Tan, Tao Li, Li Min

**Affiliations:** 1 Department of Orthopedic Surgery and Orthopedic Research Institute, West China Hospital, Sichuan University, Chengdu 610064, China; luoyuanrui2021@163.com (Y.L.); smh84028@stu.scu.edu.cn (M.S.); tanlinyun@stu.scu.edu.cn (L.T.); 2 Department of Model Worker and Innovative Craftsman, West China Hospital, Sichuan University, Chengdu 610064, China

**Keywords:** metastasis of osteosarcoma, nanoscale, drug delivery systems, treatment

## Abstract

Osteosarcoma, a predominant malignant bone tumor, poses significant challenges due to its high metastatic and recurrent nature. Although various therapeutic strategies are currently in use, they often inadequately target osteosarcoma metastasis. This review focuses on the potential of nanoscale drug delivery systems to bridge this clinical gap. It begins with an overview of the molecular mechanisms underlying metastatic osteosarcoma, highlighting the limitations of existing treatments. The review then transitions to an in-depth examination of nanoscale drug delivery technologies, emphasizing their potential to enhance drug bioavailability and reduce systemic toxicity. Central to this review is a discussion of recent advancements in utilizing nanotechnology for the potential intervention of metastatic osteosarcoma, with a critical analysis of several preclinical studies. This review aims to provide insights into the potential applications of nanotechnology in metastatic osteosarcoma therapy, setting the stage for future clinical breakthroughs and innovative cancer treatments.

## 1. Introduction

Osteosarcoma (OSA) represents a formidable challenge in oncology, predominantly afflicting children and adolescents with a slightly higher prevalence in males [1]. This aggressive malignancy, often originating in long bones, exhibits rapid proliferation and has a high propensity for metastasis, with 15–20% of patients presenting with evident metastatic spread at diagnosis—primarily to the lungs (85–90%), bones (8–10%), and, on rarer occasions, lymph nodes [2,3,4,5]. The situation is critical, as most patients likely have undetectable micrometastases at diagnosis, leading to severe morbidity and substantially lower 5-year survival rates in metastatic osteosarcoma (MOSA) compared to localized OSA [6,7,8]. The current therapeutic approach for MOSA primarily involves surgical removal of the primary tumor and apparent metastases, supplemented by neoadjuvant intensive chemotherapy. However, the high recurrence rates and the development of chemotherapy-resistant strains underscore the challenges in managing this disease [9]. This underscores the urgent need for innovative therapeutic strategies, particularly for tackling pulmonary metastasis, the most common and lethal form of OSA spread.

In this context, nanoscale drug delivery systems have emerged as potential therapeutic options in the battle against various malignancies, including OSA [10,11]. These systems, which include liposomes, micelles, and other nanoparticles (NPs), have garnered attention due to their minimal cytotoxicity to normal cells, intricate structure, and impressive drug encapsulation capabilities, offering a beacon of hope for enhancing the precision and efficacy of drug delivery in MOSA [12,13,14,15]. Chitosan nanoparticles, in particular, stand out for their biocompatibility, biodegradability, and potential for targeted drug delivery, opening avenues for personalized and less invasive treatment options [16]. Pioneering works, such as those by Tan et al., also have laid the groundwork for exploring the potential of NP therapies in MOSA [17,18,19,20,21,22]. Despite the promising advancements in nanomedicine for MOSA, the field is still in its nascent stages, with significant gaps in our understanding of optimal design, precise targeting mechanisms, and the long-term safety of these nanocarriers. This encompasses analyzing how these systems interact with osteosarcoma cells at a molecular level, evaluating their targeting efficiency within the tumor microenvironment, and determining their potential negative impact on normal cells and overall bodily functions. This review, therefore, aims to dissect the molecular-level mechanisms underlying OSA metastasis, evaluate the current landscape of nano-drug delivery systems, and critically assess their potential role in revolutionizing the treatment paradigm for MOSA.

## 2. Materials and Methods

In this narrative review, we systematically explored both the empirical and theoretical literature related to nano-based drug delivery systems in the treatment of MOSA. Recognizing the multifaceted nature of this field, our objective was to synthesize a wide range of information, ensuring relevance to the current understanding and future developments in MOSA treatment. Our literature search was meticulously executed, adhering to a structured approach influenced by the PRISMA guidelines, while being tailored to the narrative review format. This allowed us to comprehensively gather and analyze the literature pertinent to MOSA, including its mechanisms, conventional treatment modalities, and emerging nanoscale drug delivery systems.

Given the preclinical nature of the studies on nanoscale drug delivery, we concentrated on articles involving cellular and animal models. We adapted the GRADE system for evaluating the methodological quality of these studies, enabling us to critically assess and incorporate findings that provide a scientifically robust understanding of nano-based therapies in MOSA [23]. Our approach ensured the inclusion of high-quality, relevant studies, offering a holistic view that spans from the fundamental mechanisms and conventional treatments of MOSA to the innovative realm of nanoscale drug delivery systems, primarily through the lens of preclinical research.

### 2.1. Search Strategy

The literature search covered publications from January 2013 to November 2023. This period was chosen to encompass the latest developments in nano-based drug delivery systems specifically targeting metastatic osteosarcoma. 

Databases Utilized: We conducted our search through prominent scientific databases that are rich sources of medical and nanotechnology research, namely PubMed, Scopus, Web of Science, and Google Scholar.

Search Terms: Our search strategy centered around key terms relevant to our study, including “Nano-Based Drug Delivery” (MeSH term: D000090783), “Metastatic Osteosarcoma” (MeSH term: D009362), and “Nanomedicine” (MeSH term: D050997). We also incorporated terms like “Nanoparticles in oncology”, “Targeted drug delivery”, and “Osteosarcoma treatment”.

Boolean Logic: Boolean operators were utilized effectively. “OR” linked similar concepts for a broader search, while “AND” combined different aspects to enhance the specificity and relevance of our search.

Filters and Limits: Given the nascent stage of human studies in this field, our search was tailored to include preclinical studies, focusing on cellular and animal models. The search was refined with filters for full-text availability, articles in English, and publications within our defined timeframe. We specifically excluded researches on non-metastatic osteosarcoma.

Example of a PubMed Search: A representative search in PubMed might involve the query “Nano Drug Delivery” AND “Osteosarcoma” AND “metastasis“ with filters set for the specified time period, language preference, and full-text availability, focusing on cellular and animal research. 

Inclusion and Exclusion Criteria: The review selectively included preclinical studies that delve into nano-based drug delivery systems for treating metastatic osteosarcoma, encompassing both cellular and animal model research. Exclusion criteria involved non-English articles and research not pertaining to metastatic osteosarcoma or outside the set timeframe.

### 2.2. Selection Criteria

Time Frame: The literature search for our narrative review was concentrated on publications from 2013 to 2023. This decade was selected to capture the most recent advancements in nano-based drug delivery systems for metastatic osteosarcoma. The rationale for this time frame is rooted in the observation that the last ten years have seen significant progress in this field. Additionally, there has been a lack of comprehensive studies specifically addressing nano-based therapies for metastatic osteosarcoma in the earlier literature. 

Language: We focused on articles published in English to ensure wide accessibility and relevance to the global scientific community engaged in nanomedicine and oncology research. 

Study Types: Our review encompassed a variety of study designs, including experimental research, preclinical studies using cellular and animal models, and theoretical analyses. Given the nascent stage of human clinical trials in this specific area, we placed a greater emphasis on preclinical studies that provided empirical evidence and advanced theoretical understanding in the field of nanotechnology for cancer treatment. 

Content Focus: The selected articles were specifically related to the development and application of nano-based drug delivery systems in the treatment of metastatic osteosarcoma. We prioritized studies that offered insights into novel therapeutic strategies, drug delivery mechanisms, and the efficacy of nanoparticles in targeting metastatic sites. 

Population: Given the focus on preclinical studies, our review did not target a specific patient population but instead concentrated on the implications of these studies for future human application in the treatment of metastatic osteosarcoma. 

Exclusion Criteria: Articles that did not directly focus on nano-based drug delivery systems for metastatic osteosarcoma, were published outside the specified timeframe, or were not in English were excluded. Studies primarily targeting other types of cancer or non-metastatic osteosarcoma were also omitted. 

### 2.3. Article Selection

In conducting our review on nano-based drug delivery for metastatic osteosarcoma, two reviewers independently searched relevant databases and removed duplicates. The selection process began with screening article titles, followed by a detailed examination of abstracts to identify the most relevant studies. Disagreements were resolved through consensus. The final article selection focused on studies related to our topic, mainly preclinical research, due to the absence of human data. Our review primarily synthesized these findings to understand the advancements in nano-based treatments for metastatic osteosarcoma.

### 2.4. Characterization of Articles

**Figure 1:**
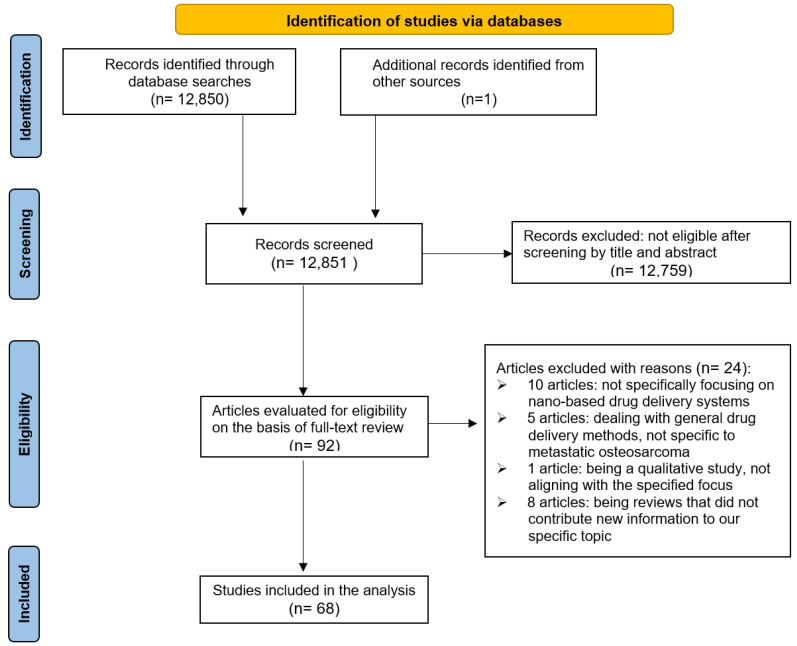
PRISMA flow chart showing the results of the search completed.

Figure 1 presents a PRISMA flow chart illustrating the results of our search process. Initially, we identified a total of 12,851 articles. After applying our selection criteria, 92 articles were deemed potentially relevant, and their full texts were obtained for further analysis. Following a comprehensive review, 24 articles were excluded for various reasons: 10 did not specifically focus on nano-based drug delivery systems, 5 dealt with general drug delivery methods not specific to metastatic osteosarcoma, 1 was a qualitative study not aligning with our focus, and 8 were reviews that did not add new information to our specific topic.

The final selection included 68 articles, with 12 experimental studies that featured a mix of in vivo and in vitro research—11 articles contained both types of experiments and 1 article was based solely on in vitro research. The 22 review articles were all topical reviews. The remaining 34 articles comprised 17 observational studies, 7 descriptive studies, and 10 studies of various methodologies pertinent to the overarching theme of metastatic osteosarcoma treatment.

### 2.5. Evaluation of Methodological Quality

To evaluate the methodological quality of the articles included in our study, we utilized the GRADE system, which is well suited for assessing clinical research. This approach allowed us to categorize the quality of evidence into four levels: high, moderate, low, or very low. These levels were determined based on factors such as study design, consistency of results, and directness of evidence. Additionally, GRADE helped us assess the strength of recommendations derived from the studies, ensuring that our review’s conclusions are grounded in scientifically robust evidence.

## 3. Results

### 3.1. Mechanism of MOSA

Osteosarcoma presents a unique set of challenges in the realm of cancer research, characterized by its distinctive genomic, epigenomic, metabolic, and microenvironmental attributes. 

#### 3.1.1. Genomic Aberrations

Osteosarcoma’s genomic profile markedly contrasts with many other malignancies. Unlike tumors such as breast cancer or melanoma, osteosarcoma rarely exhibits targetable point mutations in protein-coding genes. It predominantly features somatic copy number alterations (SCNAs) and structural rearrangements, considered pivotal to its onset and progression. Notably, there is pronounced heterogeneity in these genomic features across osteosarcoma patients, adding intricacy to therapeutic strategies [24]. Regarding metastasis, metastatic osteosarcoma samples display a heightened mutational burden and genomic instability relative to primary tumors. Genes mutated in metastatic specimens often align with the PI3K-Akt and MAPK pathways, especially during metastatic progression [25]. This complexity is accentuated by mutations in key genes like TP53, RB1, and CDKN2A, as well as amplifications in genes such as MYC and MDM2, all known to significantly influence metastasis [26]. For instance, TP53 mutations typically localize within the DNA-binding domain, impacting osteosarcoma metastasis through both suppressive and augmented functionalities. RB1 mutations, on the other hand, disrupt spliceosome function due to E2F3a upregulation [25,26]. Additionally, the SCNA landscape heralds notable amplifications like those in the 17p11.2 region, housing the TOP3A gene. This amplification is linked to the alternative lengthening of telomeres (ALT), a critical mechanism for telomere maintenance and cell longevity [24]. Structural rearrangements can also produce fusion genes, such as Rab22a-NeoF1, which propel cell migration, invasion, and metastasis [27,28]. Intriguingly, these fusion genes, by activating RhoA, influence not only cancerous cells but also neighboring non-malignant cells [28].

#### 3.1.2. Epigenetic Variations 

Osteosarcoma’s intricate nature extends beyond its genomic complexity, delving into a maze of epigenetic alterations that bolster its malignancy [29]. These modifications profoundly influence tumor development and metastatic spread, positioning them as viable therapeutic targets. Among these, DNA methyltransferases (DNMTs), particularly DNMT3A, have been pinpointed as critical in suppressing miR-149 expression via DNA methylation. This suppression subsequently activates the NOTCH1 and Hedgehog pathways, fueling osteosarcoma growth and metastasis [30]. The emerging role of long non-coding RNAs (lncRNAs) in this context is evident in molecules like THAP9-AS1. By interacting with DNMTs, THAP9-AS1 modulates methylation patterns in the SOCS3 promoter region, paving the way for JAK2/STAT3 pathway activation and, consequently, osteosarcoma progression [31]. Adding to the epigenetic intricacy is the m6A RNA demethylase, FTO, which steers mRNA demethylation [32]. Such activity has been correlated with the destabilization of KLF3 mRNA, skewing cellular behavior towards osteosarcoma advancement and metastasis [33]. Recent studies suggest DNMT-1 inhibition can enhance the sensitivity of osteosarcoma cells to specific agents like cabozantinib. This is achieved by thwarting the Notch pathway and bolstering miR-34a expression [34], thus presenting potential avenues for combinatorial therapies targeting both epigenetic and genetic facets.

#### 3.1.3. Metabolic Reprogramming

Cancer cells, including those of osteosarcoma, exhibit a characteristic metabolic adaptation to sustain their aggressive growth and survival. A prime example of this is the Warburg effect, wherein cells predominantly rely on glycolysis for energy, even in oxygen-rich conditions. Central to this metabolic shift are enzymes such as PGC1α, PKM2, ALDOA, and LDHA [35,36,37]. Specifically, miR-23b-3p suppresses PGC1α, facilitating a metabolic pivot from oxidative phosphorylation to glycolysis, consequently promoting tumor progression [38,39]. Similarly, PKM2, responsible for the pivotal glycolysis step converting phosphoenolpyruvate (PEP) to pyruvate, has attracted research attention. Recent findings suggest that PKM2’s transcriptional inhibition by IRF7 curtails aerobic glycolysis, hinting at potential therapeutic avenues [36,37]. However, osteosarcoma’s metabolic reprogramming is not limited to glycolysis. Deviations in lipid and amino acid metabolism are equally influential in metastatic dynamics. For instance, metastatic osteosarcoma cells uniquely express elevated levels of diacylglycerols. Targeting their synthesis can hinder cell migration. Furthermore, Glutaminase-1 (GLS-1), vital in glutamine metabolism, is indispensable for highly metastatic osteosarcoma cells. Its inhibition can impede metastatic advances [40,41]. Notably, CD47, pivotal for immune evasion, is influenced by amino acid uptake variations, signifying an interplay between metabolic reconfiguration and immune escape mechanisms [42].

#### 3.1.4. Dysregulated Signaling Pathways

**Figure 2:**
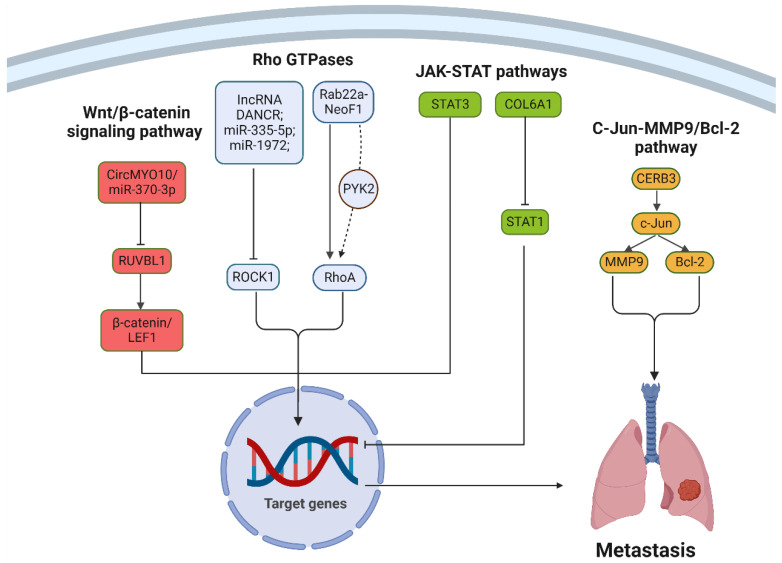
Dysregulated signaling pathways in osteosarcoma metastasis. The figure highlights key pathways, including Wnt/β-catenin, C-Jun-MMP9/Bcl-2, Rho GTPases, and JAK-STAT, driving osteosarcoma’s metastatic progression.

Osteosarcoma’s metastatic nature can be attributed, in part, to the dysregulation of numerous signaling pathways. Key among these is the Wnt/β-catenin signaling pathway, pivotal for cell fate determination, proliferation, and migration. In osteosarcoma contexts, β-catenin in the cytoplasm often misbehaves, accumulating and moving into the nucleus. Here, it partners with TCF/LEF family transcription factors, triggering Wnt-target genes like C-myc, RUNX2, and CyclinD1. This series of events accelerates epithelial–mesenchymal transition and osteosarcoma metastasis [43,44]. Another pivotal conduit is the C-Jun-MMP9/Bcl-2 pathway. Activation of the MAPK family of serine/threonine kinases leads to the phosphorylation of multiple substrates, pivotal for cell proliferation, differentiation, and migration [45,46]. In particular, the activation of c-Jun augments MMP9 and Bcl-2 expressions, bolstering the tumor’s proliferation and metastatic potential [47]. The Rho GTPases, members of the expansive Ras GTPases family, are integral for cell proliferation, progression, and migration [48]. Specifically, RhoA’s activation has been associated with osteosarcoma metastasis to the lungs. Fusion proteins like Rab22a-NeoF1 have been identified as activators of RhoA, furthering cell migration, invasion, and metastasis [49,50,51]. Lastly, the JAK-STAT pathways, housing the indispensable STAT proteins, underpin cell proliferation, differentiation, and survival [52]. Anomalous STAT3 activation in osteosarcoma cells has been linked to grim survival outcomes. Specifically, the oncogenic activation driven by STAT3 intensifies osteosarcoma’s aggressive character [53]. (Figure 2).

#### 3.1.5. Transcription Factor

In the complex landscape of osteosarcoma metastasis, transcription factors emerge as critical modulators. The NRF2 factor, for instance, orchestrates the cellular balance of ROS, steers the AMPK/mTOR autophagy signaling, and influences the Warburg effect [54]. Concurrently, RUNX proteins, DNA-binding transcription entities, preside over a multitude of genes pivotal for cellular differentiation and cell cycle evolution. Anomalously heightened levels of RUNX2—spanning its DNA, RNA, and protein manifestations—have been discernibly charted in osteosarcoma tumors [55,56]. Adding to this intricate dynamic is CREB3, a member of the leucine zipper transcription factor lineage, which can intimately bind the c-Jun promoter, thereby steering osteosarcoma evolution through genes like MMP9 and Bcl-2 [47,57]. Meanwhile, the YAP/YAZ transcription activators cast a significant shadow over osteosarcoma by regulating epithelial–mesenchymal transition (EMT) via AXL and deeply influencing cellular differentiation, fate determination, and metastatic behavior [57].

#### 3.1.6. Tumor Microenvironment 

Osteosarcoma’s intricacy is accentuated when considering its interplay with the tumor microenvironment (TME)—a rich tapestry of interactions beyond just the malignant cells. This environment encompasses immune cells, fibroblasts, and a multifaceted extracellular matrix (ECM) [58,59]. Notably, the role of tumor-infiltrating lymphocytes and macrophages is dual: they can act as both allies and adversaries to tumor progression, contingent upon their activation status [60,61]. M2-type macrophages stand out for their pro-tumor tendencies, releasing cytokines such as IL-10 and TGF-β that bolster osteosarcoma’s aggressive trajectory [61]. Cancer-associated fibroblasts (CAFs) further enrich the TME’s complexity. Characterized by markers like α-smooth muscle actin, fibroblast activation protein, and vimentin, CAFs are relentless contributors to facets like tumor expansion, invasion, drug resistance, and overall ECM reconfiguration. Through the secretion of growth factors, cytokines, and chemokines, they wield influence over cancer cell dynamics [52,62,63,64]. Furthermore, the ECM in osteosarcoma is not static; it evolves with enhanced expression of certain matrix proteins that can portend unfavorable chemotherapy outcomes and prognosis. A case in point is the extracellular matrix glycoprotein tenascin-C. Found in abundance in the TME, it liaises with the integrin α9β1 receptor, championing osteosarcoma cell migration, invasion, and metastatic progression [65].

### 3.2. Conventional Treatments for MOSA

The primary objectives in treating MOSA are to extend patient survival and alleviate associated symptoms. Despite progressive advancements in therapeutic interventions, patients’ prognosis remains uncertain. Historically, therapeutic strategies for MOSA were categorized into systemic and local treatments. The contemporary management approach integrates surgical excision of metastatic tumors, systemic chemotherapy, and, when appropriate, radiation therapy. The recent advent of targeted therapies and immunotherapies offers a more personalized treatment modality, potentially revolutionizing MOSA care (Table 1).

#### 3.2.1. Chemotherapy 

Patients diagnosed with metastatic osteosarcoma have historically relied on robust chemotherapeutic regimens to maximize tumor regression and hinder metastatic expansion. The cornerstone of these regimens has traditionally been a combination of high-dose methotrexate, cisplatin, and doxorubicin. Their efficacy in decelerating tumor growth and preventing metastasis is well documented [66]. However, selecting and administering these agents involve intricate considerations. Dosage, agent selection, and combination strategies are typically guided by the tumor’s histopathological characteristics. This dynamic interplay between tumor characteristics and treatment response mandates an adaptable approach. Clinicians frequently reassess and recalibrate treatment protocols to align with individual patient responses, always striving for a balance between therapeutic benefits and adverse effects [67]. Innovations in chemotherapeutic strategies for metastatic osteosarcoma have come to the fore recently. Specifically, the introduction of ifosfamide and etoposide into treatment regimens has garnered significant attention and optimism within the oncological sphere [68,69,70,71,72,73]. These agents have demonstrated promising results, particularly in patients who had limited or unfavorable responses to initial chemotherapy phases. Nevertheless, the challenge of chemotherapy remains. Many of these potent anti-tumor drugs, while adept at targeting osteosarcoma cells, unfortunately, also affect normal cells. Such non-specific actions can induce serious side effects, including nephrotoxicity and hepatotoxicity, among others. This underscores the essentiality of consistent patient monitoring and, when necessary, dose modulations to mitigate potential harm.

#### 3.2.2. Radiation Therapy 

Although osteosarcoma inherently resists radiation, rendering radiation therapy non-traditional as a primary intervention, its significance in the management of metastatic osteosarcoma cannot be understated. Particularly, it offers a therapeutic avenue in scenarios where surgical tumor excision is either not an option or when addressing metastatic site tumor growth [74]. With the evolution of radiation techniques, contemporary approaches such as intensity-modulated radiation therapy (IMRT), stereotactic body radiation therapy (SBRT), and proton therapy have emerged. Additionally, radiopharmaceutical interventions, such as Samarium-153 and Radium-223, enable precision-targeted high-dose radiation delivery, thereby safeguarding the surrounding healthy tissues and consequently attenuating potential side effects [75,76,77,78,79]. Nonetheless, the intrinsic radioresistance of osteosarcoma cells poses challenges to radiation’s therapeutic efficacy [80,81,82]. Considering osteosarcoma’s inclination to metastasize, especially to pulmonary regions and other remote locations, orchestrating holistic control via radiotherapy presents intricate complexities. Augmenting these challenges, extending radiation to multiple regions elevates the potential for collateral damage to adjacent healthy structures. Such unintentional damage can manifest in a myriad of complications, ranging from compromised pulmonary function and fibrotic changes to the emergence of secondary malignancies [83]. It is paramount to highlight the vulnerability of a significant subset of osteosarcoma patients: children and adolescents. Their physiologically developing organ systems amplify the risks associated with radiation-induced toxicities, necessitating an even more judicious application of this therapeutic modality.

#### 3.2.3. Surgery 

In addressing metastatic osteosarcoma, it is imperative to consider interventions for both primary and metastatic lesions. Surgical strategies at the primary site typically encompass limb-sparing surgeries and amputation. Limb-sparing surgery, aimed at preserving limb function, involves tumor excision followed by the substitution of the afflicted bone with a prosthesis or bone graft. This technique, often augmented with neoadjuvant chemotherapy to diminish the tumor’s size pre-surgery, also carries risks such as prosthetic complications and infections. Conversely, amputation guarantees the total excision of the tumor but leads to considerable functional loss and potential psychological distress [84,85,86]. When addressing metastatic lesions, often located in the lungs or other bones, surgical procedures like metastasectomy are frequently employed. This procedure is particularly effective in eliciting a complete response when the metastases are few and can be entirely excised [87]. However, metastasectomy is not without challenges: the procedure is intricate, and accompanied by high morbidity, especially when numerous metastatic sites and micrometastases are present. Furthermore, there is no absolute assurance of achieving complete resection, and the risk of future metastases persists.

#### 3.2.4. Targeted Therapies

Over the past decade, the field of oncology has experienced rapid advancements with the emergence of targeted therapies and immunotherapies tailored for metastatic osteosarcoma. These innovative treatments are designed to specifically engage with distinct aberrations in osteosarcoma cells or fine-tune the patient’s immune system to combat the tumor more efficiently. A noteworthy advancement is the introduction of agents targeting the PD-1/PD-L1 pathway [88]. This immunological pathway, pivotal to immune modulation, has been pinpointed as a prospective therapeutic target across various cancer types, including osteosarcoma. By obstructing the PD-1 and PD-L1 interaction, these agents hold the potential to bolster the immune system’s proficiency in detecting and annihilating osteosarcoma cells [88,89,90,91]. In tandem with this, there is burgeoning interest in angiogenesis inhibitors. These agents strategically hinder the development of new blood vessels within tumors, effectively depriving them of essential nutrients and stalling their growth. The underlying rationale is unambiguous: by denying tumors access to crucial nutrients, their expansion and metastatic potential can be markedly constrained [92,93,94,95,96]. Additionally, modern therapies are exploring the direct targeting of molecular anomalies specific to osteosarcoma cells. By precisely identifying and engaging with these distinct cellular markers or pathways, treatments can attain a heightened level of selectivity. This, in turn, can amplify therapeutic efficacy while concurrently diminishing inadvertent harm to healthy cells and tissues [53,97,98,99]. Yet, it is essential to recognize that these targeted interventions, like all therapeutic approaches, have inherent challenges. Paramount among these is the absolute necessity for selectivity in drug delivery. If therapeutic agents miss their specific metastatic targets, the ensuing risk is a slew of potent side effects, emphasizing the need for meticulous therapeutic delivery and monitoring.

**Table 1:** Conventional treatments for MOSA.

Treatment Approach	Key Content	Typical Indications	Advantages	Disadvantages
Chemotherapy	Combination of agents like methotrexate, cisplatin, and doxorubicin	Aggressive tumors; prepping for surgery	Shrink tumors; prevent metastatic spread	Non-selective; damage normal cells
Radiation Therapy	Advanced techniques like IMRT, SBRT, and proton therapy	Inoperable tumors; control of metastatic site tumors	Target and shrink tumors; minimize damage to healthy tissue	Osteosarcoma cells exhibit radioresistance; risk of long-term side effects
Surgery	Limb-sparing surgery or amputation; metastasectomy	Tumor is operable; metastases are limited and resectable	Completely remove tumor; limb-sparing techniques preserve function	Risk of complications, especially in the presence of multiple metastatic sites
Targeted Therapies	Agents targeting PD-1/PD-L1 pathway; angiogenesis inhibitors	Patients with specific molecular anomalies in osteosarcoma cells	More selective; tailored to individual patient’s tumor	Achieving precise delivery is challenging; potential severe side effects if not targeted correctly

### 3.3. Nanoscale Drug Delivery Systems for the Treatment of MOSA

In the dynamic realm of cancer therapeutics, nanoscale drug delivery systems (NDDS) have emerged as a cutting-edge innovation, offering a precision-focused approach to managing challenging conditions such as metastatic osteosarcoma. Central to NDDS is the deployment of nanoparticles, materials with dimensions spanning 1 to 100 nanometers, to ferry drugs to their desired destination. Engineered to amplify drug bioavailability, these systems ensure a more potent drug concentration at the target site. Their modus operandi encompasses the entrapment of therapeutic agents within these nanoparticles, which are subsequently steered to specific cells or tissues through a gamut of targeting mechanisms—both passive and active. This avant-garde approach to drug delivery has shown the potential to overhaul traditional drug delivery schematics, heralding enhanced treatment outcomes with reduced adverse reactions [100,101,102]. This review delves into the applications of the available nanoscale drug delivery tailored for combating osteosarcoma metastasis (Table 2).

#### 3.3.1. Polypeptide Nanogel

In the field of targeted drug delivery, polypeptide nanogels are fast emerging as a preferred tool. Essentially comprising chains of amino acids—polypeptides—these nanogels capitalize on their diminutive size and inherent properties to house therapeutic agents. What makes these nanoparticles particularly intriguing is their adaptability. They can be engineered to respond to specific triggers like pH alterations or changes in redox potential, enabling a measured release of the encapsulated drug [103]. The real triumph of polypeptide nanogels lies in their discerning nature. Studies underscore their proficiency in delivering the payload directly to cancer cells, bypassing healthy cells and hence mitigating unwarranted toxic effects [104]. A significant study in the arena of osteosarcoma treatment is the research by Li et al. [22], which delved into the potential of sarcoma-targeting peptide-decorated disulfide-crosslinked polypeptide nanogel (STP-NG) loaded with Shikonin (SHK), a naturally derived compound known for its antitumor activity. Their findings were both enlightening and groundbreaking. When evaluated against other groups in the study, the STP-NG/SHK complex exhibited a profound reduction in osteosarcoma’s lung metastasis. Multiple parameters—including lung morphology, weight, metastatic nodules, and H&E staining patterns—were critically assessed post-treatment. Strikingly, the STP-NG/SHK-treated group demonstrated a significant decrease in average lung weight, indicating a diminished tumor burden. This observation was further supported by a substantial reduction in the count of lung metastasis and histopathological evaluations. The latter showed that the treatment regimen with STP-NG/SHK almost entirely curtailed metastasis.

#### 3.3.2. Nanomicelles 

Within the expansive ambit of nanotechnology, nanomicelles have carved out a niche for themselves, specifically in the arena of targeted drug delivery. These nanoconstructs, measuring mere nanometers, have emerged as innovative vehicles, primarily owing to their propensity to enhance the solubility and, by extension, the bioavailability of therapeutic agents [105,106,107]. These self-assembled structures, formed from amphiphilic molecules, offer a sanctuary to hydrophobic drugs within their core, shielding them from premature degradation. More importantly, they can be engineered to actively home in on their targets; their outer surfaces can be adorned with ligands or antibodies that display a high affinity for receptors abundant in osteosarcoma cells. This ensures that the therapeutic payload reaches its intended destination, where it can be methodically released, casting a deleterious spell on the malignant cells [108]. Thus, the utilization of nanomicelles in the treatment matrix augments therapeutic outcomes and dials down systemic side effects, potentially heralding a paradigm shift in countering osteosarcoma metastasis.

Yang et al. [109] spearheaded an intriguing study where they employed Pluronic F127 to formulate nanomicelles. These were entrusted with the task of delivering [223Ra] RaCl_2_—radium dichloride, an alpha-emitting radiopharmaceutical renowned for its ability to selectively adhere to bone malignancies and metastases [110,111,112]. Clinical explorations have lauded radium dichloride for its efficacy. Remarkably, Yang et al.’s results echoed this sentiment, with their nanomicelle formulation outpacing pure radium dichloride by a hefty 20% in terms of efficiency, underscoring the promise of [223Ra] RaCl_2_-laden nanomicelles in battling osteosarcoma metastasis. This exemplifies the synergistic might of nanotechnology and targeted alpha therapy.

Similarly, Yin et al. [113] ventured into the domain of responsive drug delivery systems. They designed polypeptide micelles that respond to changes in the reducing environment. When loaded with the chemotherapeutic agent doxorubicin, these micelles showcased enhanced pharmacokinetics and improved tumor localization, with a noticeable reduction in cardiac distribution. Yin et al. pushed the boundaries further by examining the micelles’ prowess against lung metastasis in an orthotopic osteosarcoma mouse model. The results were heartening—the micelle-treated mice exhibited a drastic plunge in metastatic lung nodules, and their lungs (bearing the tumor) weighed significantly less than those of their untreated counterparts. This accentuates the potential of reduction-responsive polypeptide micelles in decelerating the onslaught of lung metastasis stemming from osteosarcoma. To conclude, the consistent strides being made in the world of nanomicelles underline their potential to redefine therapeutic strategies against the backdrop of osteosarcoma metastasis.

#### 3.3.3. Graphene Quantum Dots

In the world of nanomedicine, graphene quantum dots (GQDs) have carved a reputation as versatile nanoconstructs with profound implications for oncological applications. The essence of GQDs lies in their unique physicochemical attributes. Their expansive surface area paired with unparalleled electrical conductivity makes them prime candidates for drug delivery, potentially elevating treatment efficacies [17,114,115]. Beyond serving as mere drug carriers, their optical prowess positions them at the forefront of photothermal and photodynamic therapies. By harnessing GQDs, it is feasible to generate heat or unleash a barrage of reactive oxygen species, both aimed squarely at decimating cancerous cells. Additionally, their intrinsic fluorescence attributes augur well for bioimaging, painting a clearer cellular picture and mapping biological processes with enhanced clarity [17,18,116].

The sheer versatility of GQDs is further showcased in the pioneering work of Tung et al. [117]; researchers introduced a novel nanoscale radiosensitizer by grafting 2-deoxy-d-glucose (2DG) onto graphene quantum dots (GQD). This amalgamation, dubbed 2DG-g-GQD, merges the tumor-targeting attributes of 2DG with the radiosensitizing properties of GQDs. When tested against the 143B OS cell line, the combination of 2DG-g-GQD and radiotherapy induced a significantly elevated oxidative stress response and DNA damage, suggesting heightened therapeutic potency. Crucially, the nanoparticles demonstrated selective affinity for OS cells, ensuring targeted therapy and minimizing collateral damage. In vivo studies further solidified their potential, showcasing a marked reduction in tumor progression and a significant impediment in lung metastasis, a primary concern in OS. This innovative approach using 2DG-g-GQD offers a promising avenue for enhancing radiotherapy outcomes and curbing metastasis in osteosarcoma patients.

#### 3.3.4. Liposomes

In the vast expanse of nanomedicine, liposomes stand out as nanoscale lipid vesicles equipped with a unique structural design, allowing them to play a pivotal role in the domain of drug delivery and gene therapy. Their ability to encapsulate diverse substances positions them as adept protective shields for therapeutic agents, ensuring enhanced delivery to specified cellular or tissue targets. In addition, the inherent structure of liposomes modulates the pharmacokinetics of the drugs they harbor, paving the way for sustained drug release. This controlled release can potentially mitigate drug toxicity and improve therapeutic outcomes. Moreover, the realm of gene therapy has welcomed liposomes with open arms, leveraging their potential as vectors to ferry genetic material into cells. This prospect is particularly enticing in the context of cancer therapy [19,20,118].

Dow et al. [21] provide a compelling testament to the promise liposomes hold. In a groundbreaking study involving canines with chemotherapy-resistant osteosarcoma metastases, the team administered intravenous infusions of cationic liposomes coupled with plasmid DNA encoding canine interleukin-2 (IL-2) cDNA. Their endeavor was to gauge the potential of this treatment modality concerning immune activation, hematologic parameters, tumor responsiveness, and overall survival. The results were encouraging: not only did the treatment engender detectable IL-2 transgene expression in canine lung tissues, but it also showcased systemic immune activation hallmarked by diverse symptoms, including elevated natural killer cell activity. Impressively, a subset of the treated dogs exhibited notable regression of lung metastases. Most pertinently, these treated canines enjoyed prolonged survival times, reinforcing the potential efficacy of liposome-based therapies in combating metastatic osteosarcoma.

Another significant contribution to this narrative comes from a study that employed lipid nanoparticles to ferry edelfosine (ET), a therapeutic agent, to primary osteosarcoma tumors [119]. The results gleaned from this endeavor were profound: not only did ET encapsulated within lipid nanoparticles (ET-LN) robustly inhibit metastatic spread from the primary tumor, but it also curtailed osteosarcoma cell growth in a dose-dependent manner.

#### 3.3.5. Polysaccharide Derivative

Polysaccharide derivatives emerge as innovative biomaterials, originating from the chemical modification of native polysaccharides. Esteemed for their biocompatibility and biodegradability, these derivatives are spotlighted for their aptitude in drug delivery, particularly at the nanoscale [120,121]. Their ability to act as effective carriers allows a more targeted approach to delivering anti-tumor drugs, thereby potentially attenuating the typical systemic adverse effects seen with conventional chemotherapy. Wang et al.’s exploration [122] into this domain presents a trailblazing endeavor. The research delves into a novel nanoscale carrier designed for the delivery of astrocyte-elevated gene-1 (AEG-1) siRNA, Cs-g-PLLD-FA nanoparticles. These particles are a distinctive polysaccharide derivative, the fruition of intricate chemical processes including the click conjugation of azidized chitosan with poly (L-lysine) dendrons (PLLD) and subsequent integration with folic acid (FA). This meticulously designed system aims to proficiently transport AEG-1 siRNA into osteosarcoma cells. Evaluations conducted on osteosarcoma 143B cells in tumor-bearing mice models provided illuminating insights: not only did the nanoparticles efficiently facilitate AEG-1 siRNA transport into osteosarcoma cells, but the subsequent gene silencing led to a notable decline in tumor cell proliferation and invasion. Intriguingly, upon administration into tumor-bearing mice, these nanoparticles suppressed tumor growth and lung metastasis. The mechanistic insights revealed the silencing of AEG-1 and regulation of pivotal proteins, MMP-2/9, which are inherently involved in tumor progression. These revelations herald the potential of the novel nanoscale polysaccharide derivative as a transformative tool in metastatic osteosarcoma therapy.

#### 3.3.6. Protein-Based Nanoparticles

Protein-based nanoparticles represent an exciting paradigm shift in the arena of targeted drug delivery, capitalizing on their biocompatible and biodegradable attributes. Built from protein molecules, these nanoparticles serve as tailor-made drug delivery vehicles, unlocking the potential for precise therapeutic targeting while preserving systemic health by minimizing off-target effects [123,124,125].

Venturing into this burgeoning field, Wang et al.’s study [126] unveils the PCP-PEG-ALD nanosystem, a meticulously crafted drug delivery construct. Comprising positively charged proteins (PCP) synergized with polyethylene glycol (PEG) and the potent prodrug aldoxorubicin (ALD), this system exudes innovation. Designed to serve as an efficient carrier for ALD—a recognized chemotherapeutic agent for osteosarcoma—this nanosystem leverages physical adsorption mechanisms to encapsulate the drug, thereby potentially augmenting its therapeutic reach and impact. The resultant nanoparticles, characterized by an approximate diameter of 200 nm and a uniform morphology, boast of sustained drug release capabilities, ensuring a gradual and prolonged therapeutic effect. Significantly, the in vitro assays underscored its exemplary biocompatibility, manifesting in over 95% cell viability. However, the hallmark of the PCP-PEG-ALD system lies in its in vivo potency against osteosarcoma. Demonstrating a marked enhancement in antitumor efficacy, this nanosystem showcased dual capabilities: pronounced suppression of osteosarcoma proliferation and a significant reduction in lung metastasis. Moreover, its targeted delivery mechanism curtailed the traditional adversities of ALD, notably cardiotoxicity, further emphasizing its therapeutic promise. With enhanced therapeutic precision, reduced side effects, and potential scalability, such innovative approaches hold the key to reshaping the future of metastatic osteosarcoma management.

#### 3.3.7. Redox-Responsive Polymersomes

In the era of precision medicine, redox-responsive polymersomes herald a transformative approach to targeted drug delivery, showcasing their prowess in the treatment of osteosarcoma metastasis. These sophisticated nanocarriers, synthesized from block copolymers, have ingeniously harnessed the distinct redox gradient between cancerous and healthy tissues. Capitalizing on the heightened reducing agent concentration within tumor cells, these polymersomes disassemble, ensuring a controlled drug release in the tumor vicinity. This precision-targeted mechanism not only bolsters therapeutic efficiency but also curbs unwarranted systemic side effects, projecting these polymersomes as an avant-garde tool in osteosarcoma management [127,128,129].

Adding substance to this theory, the research by Jiang et al. [130] delves deep into the applicability of redox-responsive polymersomes in osteosarcoma therapy. They championed the IL11-PDOX nanosystem—a redox-responsive polymersome loaded with the potent chemotherapeutic agent doxorubicin (DOX). But what sets IL11-PDOX apart is its functionalization with IL-11R α-specific peptides, tailored to enhance chemotherapy specificity and efficacy against osteosarcoma. The in vitro legwork attested to this, revealing an impressive uptick in DOX internalization, apoptosis induction, and migration curtailment in IL-11R α-overexpressing 143B osteosarcoma cells when subjected to IL11-PDOX. When this insight was extrapolated to in vivo orthotopic and relapsed 143B osteosarcoma models, IL11-PDOX showcased a pronounced suppressive action against tumor proliferation and lung metastasis compared to standalone DOX treatment. Further consolidating its therapeutic validity, IL11-PDOX manifested commendable inhibitory prowess against patient-derived osteosarcoma xenografts.

#### 3.3.8. Hyaluronate-Based Nanoparticles

Hyaluronate-based nanoparticles represent a forefront innovation in targeted cancer therapy, harnessing the natural affinity of hyaluronate, a biocompatible and biodegradable polysaccharide, towards CD44 receptors, which are overexpressed in many cancer cells including osteosarcoma [131,132,133]. When engineered into nanoparticles, hyaluronate can serve as an effective vehicle for drug delivery, capable of encapsulating a wide array of anti-cancer agents. These nanoparticles can preferentially home in on osteosarcoma cells, exploiting the enhanced permeability and retention (EPR) effect commonly observed in tumor vasculature to facilitate selective drug accumulation at the tumor site [134,135]. Once at the tumor site, the nanoparticles are internalized by the cancer cells, where the encapsulated drugs are released to exert their therapeutic effects, thereby inhibiting the growth and metastasis of osteosarcoma cells. Hyaluronate-based nanoparticles offer a promising strategy to enhance the specificity and efficacy of osteosarcoma treatments, potentially reducing systemic toxicity and improving the therapeutic outcomes in patients with this aggressive form of bone cancer.

A nanoscale delivery system was developed in the study by Zhang et al. [136] called CDDPNP DOX&R848, which is a self-stabilized hyaluronic acid nanoparticle synthesized for tumor-targeted delivery in osteosarcoma immunochemotherapy. It is designed to deliver three drugs: doxorubicin (DOX), cisplatin (CDDP), and resiquimod (R848). DOX induces tumor cell apoptosis in combination with CDDP and triggers immunogenic cell death. The immune-activated tumor microenvironment created by R848 facilitates tumor-associated antigen presentation and antitumor immunity elicitation. The effect of CDDPNP DOX&R848 on inhibiting osteosarcoma metastasis was investigated in a K7M2 orthotopic osteosarcoma mouse model. The results showed that CDDPNP DOX&R848 greatly inhibited the growth of tumors and lung metastasis. This effect is attributed to the synergistic effect of chemotherapy and immunotherapy provided by the loaded drugs. CDDPNP DOX&R848 has shown promising potential as a competitive candidate for osteosarcoma immunochemotherapy.

#### 3.3.9. Graphene Oxide Nanoparticles 

In the burgeoning realm of nanomedicine, graphene oxide (GO) nanoparticles have emerged as a trailblazer, holding substantial promise for targeted drug delivery in osteosarcoma treatment. Distinguished by their unparalleled two-dimensional morphology, these nanoparticles benefit from a slew of intrinsic virtues, such as an extensive surface area, propensity for facile functionalization, and robust biocompatibility, setting them apart as a formidable weapon in the fight against cancer. One of the cornerstone features of GO nanoparticles is their ability to be tailored with specific targeting ligands. These ligands, when aligned with molecular markers prolifically expressed on osteosarcoma cells, foster precision in drug delivery. Enveloping anti-cancer drugs, GO nanoparticles are programmed to shuttle these therapeutics to the epicenter of the tumor. Once ensconced within the tumor milieu, the drugs embark on a controlled release regimen, administering therapeutic payloads squarely on osteosarcoma cells, all while sparing surrounding healthy tissues from inadvertent harm. Amplifying this therapeutic paradigm is GO’s expansive surface real estate, which underpins the loading of a copious volume of drug molecules, pushing the envelope of therapeutic outcomes. Furthermore, GO nanoparticles possess the architectural flexibility to react to external cues, be it the altered pH or temperature gradients inherent to tumor environments. This responsiveness curates a refined, tunable drug release mechanism, strengthening the therapeutic assault on osteosarcoma metastasis and cell proliferation and enhancing the prognosis landscape for afflicted individuals [137,138]. 

To crystallize this notion, the research by Lu et al. [139] offers an illuminating perspective. Their brainchild, PEG-GO-FA/ICG-Rg3—a sophisticated concoction of GO nanoparticles infused with ginsenoside Rg3, folic acid (FA), and indocyanine green (ICG)—was engineered to understand its implications on osteosarcoma metastasis. Preliminary findings painted an optimistic picture; Rg3, in its isolated form and synergistically with PEG-GO-FA/ICG, curtailed osteosarcoma cell proliferation, invasion, and migration while amplifying apoptosis and autophagy metrics. When amalgamated with near-infrared (NIR) laser treatments, the therapeutic efficacy was ratcheted up a notch, hinting at the transformative potential of this approach. Additionally, this strategic amalgamation dulled the inherent stemness of osteosarcoma-derived cancer stem cells—a significant breakthrough. Augmented by NIR laser, PEG-GO-FA/ICG-Rg3 demonstrated an appreciable inhibitory effect on osteosarcoma cell tumor growth in vivo.

#### 3.3.10. Poly(lactic-co-glycolic acid)

PLGA, standing for Poly(lactic-co-glycolic acid), represents a significant advancement in the field of biomedicine [140,141,142]. This biodegradable and biocompatible copolymer has garnered widespread attention and application in the medical and pharmaceutical sectors. Comprising two distinct monomers, lactic acid and glycolic acid, PLGA’s unique structure allows for a controlled degradation rate, which can be adjusted based on the ratio of these monomers. This adaptability is particularly beneficial for drug delivery systems. When used in drug delivery, PLGA can encapsulate a wide range of pharmaceutical agents, ensuring a sustained and controlled release over a specified duration. This controlled release mechanism can lead to improved drug efficacy, reduced side effects, and enhanced patient compliance. For instance, instead of taking a pill every few hours, a patient might receive a PLGA-based implant that steadily releases the drug over weeks or even months [143,144,145]. Furthermore, the safety profile of PLGA is well established. Once it degrades, the resulting monomers, lactic acid and glycolic acid, enter the body’s natural metabolic pathways, eventually being converted to water and carbon dioxide and excreted. This metabolic process ensures that there is no long-term accumulation of foreign substances in the body [146,147].

In the study conducted by Cao and colleagues [148], a sophisticated nanoparticulate drug delivery system was employed, specifically using Poly(lactic-co-glycolic acid) (PLGA), to encapsulate and enhance the therapeutic potential of castalin against osteosarcoma. By utilizing this nano-encapsulation technique, the team aimed to augment the solubility, stability, and permeability of castalin, optimizing its bioavailability in targeted cells. Through rigorous in vitro analyses, the PLGA-encapsulated castalin nanoparticles demonstrated potent apoptotic induction in the 143B osteosarcoma cell line, with a markedly superior efficacy compared to the native drug. Complementary in vivo experiments on LM8 xenografted mice further substantiated these findings, as notable suppression in tumor growth and metastasis was observed under nanoparticle treatment. The promising results of this study not only highlight the potential therapeutic role of castalin in osteosarcoma management but also emphasize the transformative power of nanotechnology in drug delivery and medicinal optimization.

#### 3.3.11. Chitosan Nanoparticle

Chitosan nanoparticles, stemming from the naturally occurring polysaccharide chitin found in crustacean and insect exoskeletons, are gaining prominence in the realm of drug delivery. Their inherent biocompatibility, biodegradability, and aptitude for targeted drug delivery stand them apart as potent therapeutic agents [149,150,151]. In oncology, chitosan nanoparticles take on the mantle of drug carriers, shepherding a myriad of anti-cancer agents straight to the tumor epicenter. Their intrinsic chemistry permits seamless modification, facilitating specificity to target distinct receptors prolifically found in osteosarcoma cells. Following administration, these nanoparticles employ precision targeting of the tumor, systematically releasing their drug payload and curbing tumor progression and metastasis [152,153,154]. Adding another layer of specificity, chitosan nanoparticles can be bioengineered to be sensitive to cues in the tumor milieu, such as pH shifts, paving the way for an astute drug release mechanism [155]. Such a meticulous strategy not only bolsters therapeutic efficacy but simultaneously reduces collateral damage to healthy cells, thereby painting a hopeful future for tackling osteosarcoma metastasis.

Tan et al. [16] crafted a paradigm in this domain by encapsulating dual therapeutic agents within chitosan nanoparticles: the established anti-cancer stalwart, doxorubicin (DOX), and the emerging contender, Dz13. A nuanced interplay of component concentrations birthed the Dz13-DOX nanoparticles (DDNPs), which showcased a stellar encapsulation efficiency, surpassing 91%, for both DOX and Dz13. Interestingly, these nanoparticles displayed an aggregated morphology, sometimes extending to 500 nm, and exhibited a pronounced positive Z-potential. When gauged for their impact on osteosarcoma metastasis, DDNPs eclipsed other contenders like Dz13 NPs and DOX-chitosan in tumor inhibition. Additionally, DOX delivered via traditional intraperitoneal routes paled in comparison to DDNPs. Remarkably, DDNPs not only forestalled aggressive tumor-induced bone degradation but also significantly curtailed metastatic excursions to the lungs. An icing on the cake was the benign nature of DDNPs, with mice showing no discernible adverse effects.

**Table 2:** Nanoscale drug delivery systems for the treatment of MOSA.

Nanomaterial	Loaded Moiety	Tested Models	Outcomes Reported	References
Polypeptide nanogel	Shikonin	In vitro: U-2OS, MG-63, and Saos-2 OS cells In vivo: BALB/c nude mice bearing U-2OS subcutaneous xenograft	In vitro: enhanced cytotoxicity and uptake in OS cells In vivo: notably inhibited tumor growth with reduced side effects	[22]
Nanomicelles	[223Ra] RaCl_2_	In vitro: Saos-2 OS cells In vivo: none	In vitro: 20% increased tumor-targeting efficiency vs. pure radium dichloride In vivo: none	[109]
	DOX	In vitro: 143B OS cells In vivo: 143B xenografts in nude mice	In vitro: selective inhibition of 143B OS cells through necroptosis In vivo: excellent antitumor efficacy, inhibited pulmonary metastasis with low systemic toxicity	[113]
Graphene quantum dots	2-deoxy-d-glucose	In vitro: 143B OS cells and hFOB 1.19 cells In vivo: orthotopic OS-bearing nude mice	In vitro: selectively targeted and sensitized 143B OS cells to X-ray radiation, reduced migration and invasion capacity In vivo: significantly inhibited primary OS growth, reduced the risk of metastasis	[117]
Liposomes	IL-2 cDNA	In vitro: none In vivo: dogs with spontaneous pulmonary metastatic tumors	In vitro: none In vivo: significant antitumor activity, inhibition of pulmonary metastasis, and low systemic toxicity	[21]
	Edelfosine	In vitro: MNNG-HOS and 143B OS cells In vivo: OS-induced mouse models using HOS and 143B cells	In vitro: effectively inhibited OS cell growth In vivo: remarkable antitumor efficacy, suppression of lung metastasis, and minimal systemic toxicity	[119]
Polysaccharide derivative	AEG-1 siRNA	In vitro: U-2OS cells In vivo: U-2OS cells tumor-bearing mice models	In vitro: inhibition of cell proliferation and invasion In vivo: outstanding anti-tumor efficacy, inhibited pulmonary metastasis with low systemic toxicity	[122]
Protein-based nanoparticles	ALD	In vitro: 143B OS cells In vivo: orthotopic and subcutaneous 143B OS mouse models, as well as models for cardiac injury and lung metastasis	In vitro: significantly inhibited the proliferation of human osteosarcoma cells (143B) In vivo: a strong inhibitory effect on pulmonary tumor metastasis with mitigating cardiotoxicity associated with ALD	[126]
Redox-responsive polymersomes	DOX	In vitro: 143B OS cells In vivo: mouse model of orthotopic 143B OS, relapsed OS and PDX OS	In vitro: induced apoptosis and hindered 143B OS cell proliferation In vivo: significantly inhibited tumor growth and lung metastasis, while sparing systemic toxicity	[130]
Hyaluronate-based nanoparticles	DOX,CDDP, R848	In vitro: OS cell lines, moDCs, and human primary T cells In vivo: 143B tumor-bearing mice model	In vitro: effectively delivered drugs and siRNA to OS cells, promoting T cell activation In vivo: significantly inhibited tumor growth and prolonged mice survival	[136]
Graphene oxide nanoparticles	Ginsenoside Rg3, ICG	In vitro: Saos-2 and MG-63 OS cells In vivo: MG-63 tumor-bearing BALB/c nude mice	In vitro: enhanced cytotoxicity in OS cells In vivo: significantly suppressed tumor growth in MG-63 tumor mice	[139]
Poly(lactic-co-glycolic acid)	castalin	In vitro: 143B OS cells In vivo: LM8 xenografted mice	In vitro: reduced cell viability in 143B cells and induced apoptosis effectively In vivo: showing a more pronounced reduction in tumor volume	[148]
Chitosan nanoparticle	DOX, Dz13	In vitro: Saos-2 cells In vivo: Saos-2 tumor growth in mice	In vitro: successfully reduced Saos-2 viability by up to 80% and maintained their cell-kill efficiency In vivo: inhibited Saos-2 tumor growth and spread in mice more effectively	[16]

OS, osteosarcoma; DOX, doxorubicin; ALD, aldoxorubicin.

## 4. Discussion

The significance of nanoscale drug delivery systems (NDDS) in metastatic osteosarcoma (MOSA) treatment is paramount, addressing critical challenges in current therapeutic strategies. Traditional treatments often fall short in targeting the metastatic nature of osteosarcoma due to limitations in drug specificity and systemic toxicity. NDDS, with their ability to precisely target tumor sites and control drug release [156,157,158,159,160], present a groundbreaking approach in MOSA management. Nanoscale systems, including liposomes, nanomicelles, and graphene quantum dots, offer enhanced drug bioavailability, ensuring higher concentrations of therapeutic agents are delivered directly to the tumor site. This targeted approach not only maximizes the therapeutic efficacy but also significantly reduces adverse effects commonly associated with chemotherapy. Particularly in MOSA, where the tumor’s aggressive and metastatic nature poses a formidable challenge, the precision of NDDS can lead to more effective treatment outcomes [160]. Moreover, the adaptability of these nanoscale systems to respond to specific environmental triggers within the tumor microenvironment, such as pH changes or redox potential, enables a controlled and sustained release of drugs. This not only enhances the effectiveness of the treatment but also minimizes the potential harm to healthy cells. The ability of NDDS to encapsulate and protect drugs from premature degradation further contributes to their effectiveness [161]. Another key aspect of NDDS in MOSA therapy is their potential for personalized treatment. Given the heterogeneous nature of osteosarcoma, treatments that can be tailored to the individual’s specific tumor profile are invaluable. NDDS can be designed to target specific molecular markers unique to the patient’s tumor, paving the way for more personalized and effective cancer care.

Nevertheless, the journey of integrating these systems into the therapeutic paradigm of MOSA remains intricate. While the potential of these systems is undeniable, it is paramount to critically appraise the inherent challenges. The complexities of long-term safety evaluations, potential emergence of drug resistance, challenges in scalability, and the intricacies of manufacturing underscore the imperative need for continuous research and innovation. 

Additionally, as this review predominantly discusses preclinical findings, we acknowledge the unpredictability of transitioning from bench to bedside. Clinical successes may not always mirror preclinical triumphs, and potential unforeseen obstacles might arise in real-world scenarios. As the field of NDDS in the treatment of metastatic MOSA is still in its nascent stages, it is essential to acknowledge the current lack of extensive clinical trials and related data in this area. As the scientific community continues its investigations, we anticipate more robust clinical data in the foreseeable future. As we look ahead, a multidisciplinary collaboration, harnessing expertise from oncology, nanotechnology, and pharmacology, is undeniably the cornerstone for future breakthroughs.

## 5. Conclusions

In conclusion, our review elucidates the promising horizon of NDDS in managing MOSA. We have highlighted how traditional therapeutic modalities often fall short in specificity and control, leading to systemic toxicity and limited efficacy in targeting metastatic sites. The detailed exploration of various NDDS, including liposomes, nanomicelles, graphene quantum dots, and more, showcases their potential in enhancing drug bioavailability, reducing adverse effects, and offering a more targeted approach to MOSA. These systems not only exhibit improved tumor site targeting but also offer adaptable release mechanisms responsive to specific tumor microenvironmental triggers. Our review underscores the revolutionary impact these nanotechnologies could have in MOSA treatment, presenting a potential paradigm shift in oncological therapeutics. However, the translation of these promising preclinical findings into clinical practice demands further research, addressing challenges in scalability, safety, and regulatory approval. This review sets a foundation for future exploration and development in this exciting field, with the hope of translating these advanced therapeutic strategies from bench to bedside, offering new hope for patients afflicted with MOSA.

## Data Availability

Not applicable.
